# Intranasally administered *in situ* gelling nanocomposite system of dimenhydrinate: preparation, characterization and pharmacodynamic applicability in chemotherapy induced emesis model

**DOI:** 10.1038/s41598-017-10032-7

**Published:** 2017-08-30

**Authors:** Sara S. Barakat, Maha Nasr, Rania F. Ahmed, Sabry S. Badawy, Samar Mansour

**Affiliations:** 1 Department of Pharmaceutics and Industrial Pharmacy, Faculty of Pharmacy, Misr International University, Cairo, Egypt; 2 Department of Pharmaceutics and Industrial Pharmacy, Faculty of Pharmacy, Ain Shams University, Cairo, Egypt; 3 Pharmacology Department, National Research Centre, (ID: 60014618), Dokki, 12622 Giza Egypt; 4 Pharmaceutical Technology Department- German University in Cairo, Cairo, Egypt

## Abstract

The aim of the current manuscript was to test the applicability of a nanocomposite system of penetration enhancer vesicles (PEVs) within polymeric *in situ* forming gel network composed of poloxamer and hyaluronic acid for the intranasal delivery of the antiemetic dimenhydrinate (DMH). PEVs were prepared using phospholipids and labrasol/transcutol/PEG 400 as penetration enhancers, and characterized for entrapment efficiency (EE%), particle size, zeta potential and morphology. The nanocomposite *in situ* forming gel system was characterized for its sol-gel temperature, viscosity and mucoadhesiveness, and was pharmacodynamically tested on a cisplatin induced emesis model in rats in terms of food, water, kaolin intake and stomach weight content. The selected PEVs formula displayed EE% of 83% for DMH, particle size of 121 nm and a surface charge of 0.83 mV. The selected nanocomposite *in situ* gelling formula showed a viscosity of 2.13 Pa.S, mucoadhesive force of 0.62 N and DMH controlled release over 6 hours. The pharmacodynamic study showed the superiority of the nanocomposite *in situ* gelling formula; being administered at a lower dose than the oral marketed formula. The described nanocomposite system proved to be successful for the intranasal delivery of DMH, thus presenting a promising delivery modality for similar antiemetics.

## Introduction

**Figure 1:**
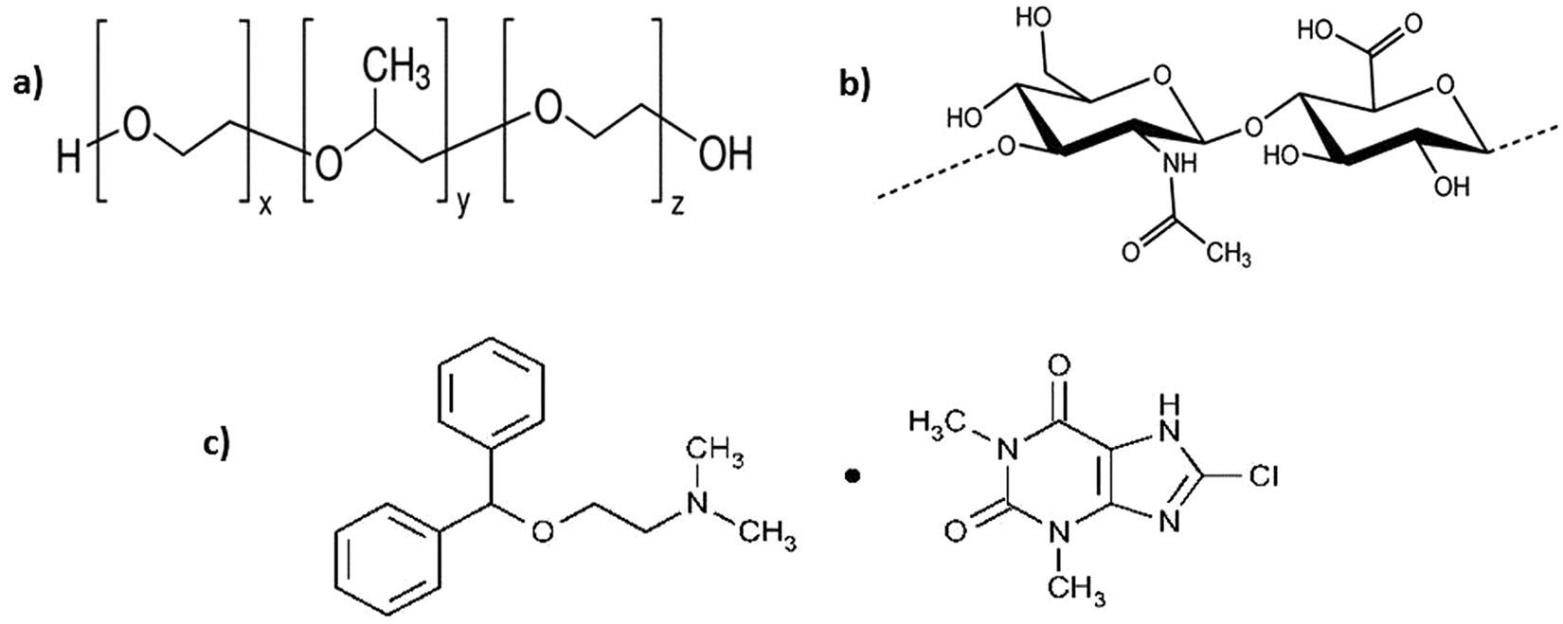
Chemical structure of (**a**) Poloxamer (**b**) Hyaluronic acid (**c**) Dimenhydrinate.

The current advances in technology have led to the development of nanoparticles which are structures of sizes ranging from 1 to 1000 nm. Nanocarriers with optimized physicochemical and biological properties can be successfully used as delivery tools for several bioactive compounds, as they are uptaken by cells more easily than larger molecules. They also increase the solubility of drugs, improve their bioavailability, provide a prolonged drug release and offer targeted drug delivery^1^. Penetration enhancer vesicles “PEVs”; which are composed of a penetration enhancer intermingled with phospholipids have recently presented themselves as a new versatile vesicular nanocarrier with wide variety of applications^2–7^; namely for topical and transdermal delivery purposes. With the emergence of several functional materials that could be used in drug delivery, the term “nanocomposite” was introduced, which refers to a class of hybrid materials consisting of dissimilar components of different properties combined in one system^8^. Poloxamer; which is a surface-active block copolymer of polyoxyethylene and polyoxypropylene (Fig. 1a) is known for its excellent compatibility with other chemicals, good release properties of drugs, and most importantly, its thermoreversible nature^9^. Another biocompatible material is hyaluronic acid (HA), which is a natural anionic polysaccharide (Fig. 1b) known for its bioadhesive and controlled drug release properties as well^10, 11^. The combination of a filler in the nanoscale “PEVs in our case” within a material of different nature “hyaluronic acid based *in situ* gelling system of poloxamer” is expected to confer different properties to the newly created system, by preservation or augmentation of the merits of both^8^.

The intranasal route has been recently explored as a non-invasive route for rapid delivery of drugs directly from the nasal mucosa to the systemic circulation and to the brain for treating central nervous system disorders^12^. This is attributed to the nasal anatomy, which provides high surface area with high blood flow, and where the cells of the olfactory region in the nasal mucosa extend up into the cranial cavity. Upon nasal administration, the delivery system comes in contact with the mucosa, leading to the transportation of the drug to the systemic circulation and to the brain, skipping the blood brain barrier (BBB) and achieving rapid response^13, 14^. The intranasal delivery of antiemetics has been investigated in the treatment of chemotherapy-induced nausea and vomiting, to achieve rapid drug absorption either systemically or by direct transport to the brain^15–17^.

Dimenhydrinate (DMH) (Fig. 1c) is an antiemetic drug used for the prevention and treatment of nausea and vomiting associated with motion sickness and induced by chemotherapy^18, 19^, through the inhibition of histamine H1 and muscarinic receptors, and the antiemetic effect of DMH is mainly due to its regulatory potential which affects the vestibular nuclei and the closely associated centers in the brainstem^20^. DMH acts mainly on the central vestibular system through the inhibition of histamine receptor and cholinergic receptor functions in the vestibular nuclei and vomiting center^21^. The oral administration of DMH suffers from extensive hepatic metabolism, and tends to be discharged by vomiting^18^. In addition, its onset of action is 60 min after oral administration, which renders it unsuitable for treatment of emergency situations^22^. These aforementioned properties nominate DMH as a possible model drug for intranasal delivery.

Therefore, the aim of this work was to prepare a nanocomposite *in-situ* forming gelling of DMH, created by the incorporation of PEVs loaded with the drug within a mucoadhesive *in situ* gelling system based on poloxamer and hyaluronic acid, to achieve rapid onset with sustained delivery potential to enhance the therapeutic efficacy of DMH. The efficacy of the nanocomposite system was compared to that of the mere vesicles and the *in situ* forming gel in a chemotherapy-induced emesis animal model.

## Materials and Methods

### Materials

Dimenhydrinate was kindly supplied by Alkahira Pharmaceuticals & Chemical Industries Company, Cairo, Egypt. Poloxomer 407 (P407) and Poloxomer 188 (P188) were kindly gifted by BASF pharmaceutical company, Germany. Sodium hylauronate (HA) was kindly gifted by Freda Biopharmaceuticals, China. Sodium chloride (NaCl), Sodium dihydrogen orthophosphate 1 – hydrate, Polyethylene glycol 400 (PEG), Disodium hydrogen orthophosphate anhydrous, acetic acid, methanol, chloroform and diethyl ether were purchased from El-Nasr Chemical Company, Cairo, Egypt. Mucin (Crude porcine mucin), gum Arabic and kaolin were purchased from Sigma, Aldrich. Chitosan (CS) (Chitoclear, grade fg 95LV, molecular weight below 150 kDa) was kindly gifted by Primex company, Iceland. Dialysis membrane (Spectra/Por) 12.000–14.000 molecular weight Cut off was purchased from Spectrum Laboratories USA. Capryl-caproyl macrogol 8-glyceride (Labrasol®) and 2-(2-ethoxyethoxy) ethanol (Transcutol®) were kindly gifted by Gattefosse’ Company, France. Phosphatidylcholine (Epikuron 200) was kindly provided by Cargill Texturizing solutions, Deutschland GmbH & Co., Hamburg, Germany. Acetyl Uranil (Uranyl acetate -2- hydrate) was purchased from Allied signal, Riedel- dehaen, Germany). Dramenex® tablets were purchased from Alkahira Pharmaceuticals & Chemical Industries Company, Cairo, Egypt. Cisplatin was purchased from Oncotec Pharma Produktion GmbH, Dessau, Germany.

### Preliminary experiments for selection of the most suitable mucoadhesive *in situ* forming gelling system of DMH

Before conduction of the preliminary studies, all experimental procedures were approved by the ethical committee of faculty of pharmacy, Ain Shams University (approval number ASU 94), and were conducted according to the guidelines and regulations dictated by this protocol.

Formulations containing different concentrations of the thermosensitive polymers P407 and P188 were prepared using the cold method^17, 23, 24^. The composition of the prepared formulations is shown in Table (1). Briefly, the *in situ* gelling formulations were prepared by dissolving the drug (with or without PEG 400) in phosphate buffer saline (PBS) pH 7.4 at room temperature followed by sonication (Transsonic, model TI-H 5, Germany). The thermosensitive polymers P407 and P188 were then added to the buffer in cold water bath on a magnetic stirrer (Jenway, England) followed by storage in the refrigerator with periodic stirring till homogenous formulations are obtained. In order to preliminary assess the effect of drug amount on the formulation of the *in situ* forming gelling system; variation of the amount of drug from 50 mg to 75 mg and 100 mg was carried out as shown in Table (1). The amount of incorporated DMH was then escalated to 200 mg.

**Table 1:** Composition of the prepared *in situ* gelling systems of dimenhydrinate and their gelation temperature.

Formula code^*^	Amount of Drug (mg)	Percentage of Poloxomer 407 (%w/w)	Percentage of Poloxomer 188 (%w/w)	Volume of Buffer PBS pH7.4 (ml)	Mucoadhesive agent and its concentration (%w/v)		Sol- gel temperature (°C) Mean ± S.D
					HA	CS	
F1	50	18	—	8.15	—	—	>60 °C
F2	75	18	—	8.125	—	—	
F3	100	18	—	8.1	—	—	
F4	200	18	—	8	—	—	ND**
F5	200	17	—	7.6	—	—	28.4 ± 1.7
F6	200	18	—	7.5	—	—	23.3 ± 0.4
F7	200	17	13	6.3	—	—	41.1 ± 3.4
F8	200	18	13	6.2	—	—	34.8 ± 0.3
F9	200	19	12	6.2	—	—	30 ± 0
F10	200	20	11	6.2	—	—	30.3 ± 0
F11	200	9	25	5.9	—	—	>60 °C
F12	200	12	20	6.1	—	—	
F13	200	15	15	6.3	—	—	
F14	200	18	13	5.7	—	0.01	ND**
F15	200	18	13	5.5	—	0.02	
F16	200	18	13	6.08	—	0.05	
F17	200	18	13	5.88	—	0.1	
F18	200	18	13	5.12	—	0.2	
F19	200	18	13	4.07	—	0.5	
F20	200	19	12	5.5	—	0.02	
F21	200	20	11	5.95	—	0.01	
F22	200	20	11	5.88	—	0.02	
F23	200	20	11	6.05	1.5	—	
F24	200	18	13	6.15	0.5	—	39 ± 2
F25	200	18	13	6.1	1	—	38 ± 1.8
F26	200	19	12	6.15	0.5	—	35 ± 2.5
F27	200	19	12	6.1	1	—	34 ± 0.5
F28	200	20	11	6.15	0.5	—	29.4 ± 0.5
F29	200	20	11	6.1	1	—	30 ± 0.5

^*^PEG 400 (0.5 ml) was included in all formulae except F1-F4.

^**^Not determined since the formulae showed precipitation of DMH.

HA: Sodium hylauronate.

CS: Chitosan.

After reaching DMH *in situ* gel formulae which undergo gelation at the physiological temperature, preliminary experiments were conducted for the incorporation of mucoadhesive using either chitosan (CS) or hyaluronic acid (HA) as also shown in Table (1). The mucoadhesive substances were added to the *in situ* gelling formulations showing acceptable gelation temperature.

For mucoadhesive formulations prepared using CS, the chitosan solutions were prepared by dissolving it in 0.1 N acetic acid. CS solutions were then added to the dissolved drug with PEG 400 in the buffer PBS (pH 7.4) at room temperature with sonication, to achieve different final CS concentrations. The addition of P407 and P188 then followed in cold water bath. The solutions were stored in the refrigerator with periodic stirring till homogenous.

For mucoadhesive formulations prepared using HA, formulae were prepared by dissolving the drug, PEG 400 and different concentrations of HA in the buffer PBS (pH 7.4) at room temperature with the aid of sonication, according to the concentrations shown in Table (1). After the mucoadhesive polymer HA was dissolved, the poloxomer P407 and P188 were added to the formulations. The formulations were left in a cold water bath with magnetic stirring till complete dissolution of poloxamer, followed by refrigeration storage with periodic stirring.

### Measurement of the sol–gel transition temperatures (Tsol–gel) of the prepared *in situ* gelling systems

The temperature at which the prepared mucoadhesive thermosensitive *in situ* gels with different combinations of P407, P188 and the mucoadhesive agent turn into gel (Tsol–gel) was measured using the method described by several authors^17, 25, 26^. This was done to select the most appropriate combination of P407, P188 and the mucoadhesive agent that can be used for incorporating the DMH loaded PEVs. This was performed by taking a 2 ml aliquot of each formulation in a test tube which was then sealed by a parafilm and placed in a thermostatically controlled water bath (Thermo Haake model SWB25, USA). The temperature of the water bath was adjusted at 20 °C and then gradually increased in increments of 3 °C (0.5 °C in the region of the sol-gel temperature). The formulae were allowed to equilibrate at each new temperature for 2 minutes. Then the gelation temperature was determined when the meniscus remain immobile by tilting the test tube 90°.

### Preparation of DMH loaded PEVs

After preliminary screening of the optimum preparation conditions for DMH PEVs^27^, two DMH-loaded PEVs formulae were prepared; named (P) and (V), according to the composition described in Table (2). The PEVs formulae were prepared using the reversed phase evaporation technique (REV)^28^. The phospholipid was dissolved in chloroform: methanol mixture (2:1, v/v) in a round bottomed flask, followed by evaporation of the organic solvent mixture using a rotary evaporator at 40 °C (Rotavapor R-210/215, Buchi, Switzerland) resulting in the formation of lipid thin film on the inner wall of the flask. The film was re-dissolved in 12 ml diethyl ether, followed by the addition of certain volume of phosphate buffer saline pH 7.4^28, 29^ containing 200 mg DMH solubilized in 0.5 ml PEG 400, with the penetration enhancers labrasol and transcutol included in the buffer at a ratio of 1:1 v/v. The system was swirled by hand, and the formed emulsion was then placed in the rotary evaporator where the organic solvent was removed under reduced pressure to fully remove the organic solvent followed by the addition of the remaining volume of the phosphate buffered saline. The resulting dispersion was then sonicated (Transsonic model: TI-H 5, Germany) at 40 °C for one hour to reduce the particle size of the vesicles^30^.

**Table 2:** The composition of DMH vesicles and nanocomposite *in situ* gelling systems and their corresponding gelation temperatures.

Formula code^*^	Total volume of PBS pH 7.4 in the formula (ml)	Amount of Penetration Enhancer (ml)			Amount of phospholipid (mg)	Percentage of P407 (%w/v)^**^	Percentage of P188 (%w/v)^**^	Sol- gel temperature (°C) Mean ± S.D.
		Labrasol	Transcutol	PEG 400				
P	6.5	1.5	1.5	0.5	900	—	—	—
P1						19	12	>37
P2						31	—	ND^***^
P3						—	31	>37
V	7.5	1	1	0.5	600	—	—	—
V1						19	12	29.33 ± 0.58
V2						18	13	>37
V3						17	13	>50
V4						15	15	>50
V5						18.5	11.5	32.83 ± 1.04
V6						18.75	11.25	35.67 ± 0.29

^*^All formulae were prepared using 200 mg DMH. ^**^All poloxamer containing formulae also contained 1% HA. ^***^Not determined owing to the high viscosity of the gel.

### Separation of unentrapped DMH from the prepared PEVs

Purification of PEVs from the non-encapsulated drug was done by exhaustive dialysis, in which PEVs formulae were placed in a dialysis tubing (Dialysis membrane Spectra/Por 12.000–14.000 molecular weight)^31^. The dialysis was carried out against 1000 ml distilled water for 2 hours^7^, which was found appropriate for the removal of the non-entrapped DMH in the medium.

### Characterization of the prepared PEVs

#### Determination of DMH entrapment efficiency percentage in the PEVs

To determine the amount of DMH entrapped in the dialyzed vesicles, the vesicles were disrupted using methanol^32^. Five hundred microliters of PEVs were mixed with 4.5 ml of methanol to obtain a clear solution, which was covered well with a parafilm to prevent methanol evaporation. The concentration of DMH in methanol was determined spectrophotometrically at 278 nm after appropriate dilution (Shimadzu, UV1650, Europe). The entrapment efficiency (EE%) was calculated through the following relationship^33, 34^:

1 $${\rm{Entrapment}}\,{\rm{Efficiency}}\,{\rm{Percentage}}=\frac{{\rm{Entrapped}}\,{\rm{drug}}\times {\rm{100}}}{{\rm{Total}}\,{\rm{drug}}}\,$$

### Determination of the particle size and zeta potential of PEVs

The size, polydispersity index (PDI) and charge of the prepared DMH PEVs were determined using Zetasizer (Nano ZS 3600, Malvern Instruments Ltd., WorcesterShire, UK)^6, 7, 35, 36^ after dilution 1:100 with deionized water at 25 °C and detection angle 173° after equilibration for 120 seconds. The refractive index was set to 1.33.

### Stability study

The prepared DMH loaded PEVs were stored for three months at refrigeration temperature 2–8 °C. After the three months storage period, the samples were inspected visually for their homogeneity and consistency. The particle size, zeta potential and polydispersity index (PDI) of the PEVs were also re-measured.

### Determination of the morphology of PEVs using transmission electron microscope (TEM) prior to incorporation in the *in situ* gelling system

The selected PEVs formula (V) (to be incorporated in the selected *in situ* gelling system) was tested for morphology by transmission electron microscope (TEM, model JEM-100 S, Joel, Tokyo, Japan)^37^. The analysis was done by depositing one drop of the diluted sample on a film coated 200-mesh copper grid, followed by uranyl acetate staining (1%) and drying^32^. Before examination any excess fluid was removed with filter paper.

### Preparation of DMH nanocomposite *in situ* gelling systems

After selecting the most appropriate concentrations of poloxamers for preparation of the *in situ* gelling systems (showing gelation at the physiological body temperature range), the prepared PEVs formulae were incorporated in the thermosensitive *in situ* forming gelling systems after the preparation of the PEVs formulae using the reversed phase evaporation technique (REV) as described in the PEVs preparation section. Hyaluronic acid 1% w/w, in addition to poloxamers P407 and P188 were incorporated within the PEVs dispersion followed by magnetic stirring (Jenway model 1000, England) in ice bath and overnight refrigeration till the dissolution of poloxamers, creating nanocomposite *in situ* gelling systems. The composition of the nanocomposite DMH *in situ* gelling formulae is described in Table (2).

### Characterization of DMH nanocomposite *in situ* gelling systems

#### Measurement of the sol–gel transition temperatures (Tsol–gel) of selected formulations

After formulating the nanocomposite systems by incorporating DMH loaded PEVs with different composition in the *in situ* gelling systems of various compositions, their gelation temperatures were also measured as previously described for the *in situ* gelling systems.

#### Measurement of the viscosity of selected formulations

The selected nanocomposite formulation of DMH showing a sol-gel temperature in the physiological range was examined for its viscosity, and compared with the viscosity of its corresponding PEVs formula. Viscosity measurements were done both at 25 °C representing the room temperature and 37 °C representing the body temperature. The viscosity measurement was done using a rheometer^38^ (model Anton Paar Gmbh, 3ITT, Austria) at a fixed shear rate of 50 s^−1^.

### *In vitro* release of DMH from selected formulations

Using a modified rotating basket method^33, 34^, the release of DMH from the selected nanocomposite formulation showing sol-gel temperature in the physiological range and its PEVs and gel counterparts was determined at 37 °C and 50 rpm^22, 39^. One ml of the formula was placed in a glass cylinder (2.5 cm diameter) with a cellulose membrane tied to one end, and attached to the metallic shaft of the dissolution apparatus (replacing the basket) at the other end^40^. The cylinder was then lowered to touch the surface of the dissolution medium (500 ml of phosphate buffer pH 6.8). At different time intervals namely (15, 30, 45, 60, 120, 180, 240 and 300 min), 3 ml samples were withdrawn and replaced by fresh buffer. The released amount of DMH was determined at 278 nm^22^.

### Measurement of the mucoadhesive strength of the selected formulations

The texture analyzer CT3 setup for tension test (TAXT plus, Stable Microsystems, UK) was used for examining the mucoadhesive strength of the selected nanocomposite *in situ* gelling formulation and its PEVs counterpart as described by several authors^39, 41, 42^. The method depends on measuring the force required to detach the formulations from a mucin disc prepared by using crude porcine mucin which was compressed by a single punch tablet press (10 mm diameter die). The formulae were maintained at a temperature 37 °C prior to the initiation of the experiment.

A fixed amount of the formula was kept on the lower platform while a mucin disc was attached to the upper probe of the equipment. At a speed of 1 mm/s the upper probe was then lowered touching the surface of the gel and a force of 0.1 N was applied for 3 min ensuring complete contact between the mucin disc and the gel. When the trigger value was reached the total download distance moved by the probe represents the deformation which was set up to 20 mm.

### Stability of the selected formulation

The selected nanocomposite *in situ* gelling formulation was stored in the refrigerator for three months. After the three months storage period, the sample was visually inspected for its homogeneity and consistency. The gelation temperature of the formula was re-measured.

### Pharmacodynamic study using a chemotherapeutically induced emesis model

Rats and mice respond to emesis stimulating factors by induction of pica i.e. the consumption of a substance without nutritional value such as kaolin. Thus, pica has been suggested to be analogue to vomiting in species that do not vomit^43^. Cisplatin is a cancer chemotherapeutic agent which was reported to induce pica in a species endowed with this reflex, manifested by an increase in kaolin intake, a decrease in the food and water intake and a decrease in the gastric emptying, resulting in an increase in the weight of the stomach content. The experimental procedures were approved by the ethical committee of faculty of pharmacy, Ain Shams University (approval number ASU 94).

Six groups of adult male albino Wistar (130–150 gms) rats were used. Each group included 8 rats intraperitonially (i.p.) injected by cisplatin as a pica inducing drug. Kaolin was mixed with 1% gum Arabic to prepare a thick paste of kaolin (China clay). It was further extruded, dried at room temperature, and then cut into pellets to form rods with a similar size to the normal food pellets. For a 3 day habituation period prior to the start of the experiment each animal was supplied by food, water and kaolin after being placed in an individual cage, and their food, water and kaolin consumption was assessed. The groups were divided as follows:

Group I: **Normal “control” group** (given distilled water orally using the oral tube without administration of cisplatin injection).

Group II: **Induced “reference” group** (given distilled water orally by the oral tube before i.p. cisplatin injection).

Group III: Standard **group** (given crushed Dramenex® marketed product orally using the oral tube 15 min before i.p. cisplatin injection, at a dose of 10 mg/kg equivalent to 1.3–1.5 mg DMH per rat).

Group IV: PEVs **formula test group** (Intranasal administration of 25 µl of the PEVs formula in each nostril before i.p. cisplatin injection by 15 min, corresponding to a dose of 1 mg DMH per rat).

Group V: **Mucoadhesive thermosensitive** ***in situ*** **gelling group** (Intranasal administration of the *in situ* gelling system corresponding to a dose of 1 mg DMH per rat).

Group VI: **Nanocomposite formula test group** (Intranasal administration of the DMH *in situ* gelling nanocomposite formula before i.p. cisplatin injection by 15 min, corresponding to a dose of 1 mg DMH per rat).

### Histological study on the selected formulations on rat nasal mucosa

Nasal histopathological examination was carried out for two groups of male albino rats weighing 130–150 g. A volume of 25 µl of the selected mucoadhesive thermosensitive *in situ* gel and nanocomposite formulae was administrated in each nostril of the rats of each group using a micropipette, corresponding to a dose of 1 mg DMH. Autopsy samples were taken from the nose of scarified rats after 24 hours of intranasal formula treatment^17^. The samples were then fixed in 10% formal saline for twenty four hours, followed by washing, drying and sectioning at 4 microns thickness using a sledge microtome. Tissue sections were stained by hematoxylin and eosin stain and examined using optical microscopy^44^ (Axiostar plus, Zeiss, New York, USA).

### Statistical analysis

The obtained data was statistically analyzed using Graph pad Instat program. Data were expressed as the mean ± standard deviation (S.D.), or as mean ± standard error of the mean (S.E.) Comparison of the mean values was done using one way analysis of variance (ANOVA), followed by Tukey – Kramer Multiple Comparisons Test. Statistical significance was set at p-value ≤ 0.05.

## Results and Discussion

### Preparation of the thermosensitive *in situ* forming gels of DMH

DMH used at various amounts reaching 100 mg (F3) were completely soluble in the used buffer PBS (pH7.4) without any solubilizing assistance. In order to elevate the therapeutic amounts of the drug reaching the brain, the amount of drug to be loaded in the *in situ* forming gelling system was increased to 200 mg (F4), however, the drug was not dissolved and precipitates of the drug were apparent, which necessitated the addition of a solubilizing agent. The chosen solubilizing agent was PEG 400^18^, in which 0.5 ml of PEG was sufficient to solubilize the 200 mg DMH. Hence, it was included at this volume in formulae (F5-F29).

### Measurement of the sol–gel transition temperatures (Tsol–gel) of the *in situ* forming gels of DMH

As evident in Table (1), formulae (F1-F3) prepared using 18% poloxomer 407 (P407) with variable drug concentrations gave gels at very high temperatures (>60 °C). The high gelation temperature exhibited by the *in situ* gelling formulae (F1-F3) containing poloxomer 407 was also previously reported by Mansour *et al*.^9^, who indicated that gelation at the physiological temperature (30–36 °C) was not achieved by P407 (up to 20%) nor P188 (up to 30%) alone, and it is recommended to use combination of both P407 and P188 in different ratios for preparation of the *in situ* forming gelling systems.

The addition of PEG 400 significantly decreased the gelation temperature of *in situ* gelling systems prepared using poloxamer P407 only (P < 0.05), in which formulae (F5 and F6) prepared solely using poloxamer P407 (expected to gel at high temperatures as F1-F3) exhibited gelation temperatures of 28.4 ± 1.7 and 23.3 ± 0.4 respectively. This matched the findings of Ricci *et al*.^45^, who indicated that the addition of PEG caused a decrease in the sol-gel transition temperature owing to its hydrophilic nature, enabling it to bond with water molecules and hence, decreasing the quantity of free water molecules. Therefore, the gelation process occurs at a lower temperature as these systems have less water molecules that are able to form bonds with the polymer molecules.

In order to obtain formulations with desirable gelation range, variation of the ratio of poloxamer P407 to P188 was achieved. As evident in Table (1), increasing the ratio of poloxamer P407 from 17% to 20% significantly decreased the gelation temperature (P < 0.05). This was clear in (F7) with 17% P407 showing gelation at 41.1 ± 3.4 °C while the gelation temperature significantly decreased to 30.3 ± 0 °C in (F10) with 20% P407 (p < 0.05). Only the formulations (F8, F9, F10) with P407/P188 ratio (18/13), (19/12) and (20/11) showed sol-gel transition temperatures in the physiological range, while other combinations (F11, F12, F13) with P407/P188 ratio (9/25), (12/20) and (15/15) displayed extremely elevated sol-gel transition temperatures, hence, they were excluded. The significant decrease in the gelation temperature upon increasing the ratio of poloxamer P407 from 17 till 20% in formulae (F7, F8, F9 and F10) could be attributed to the higher number and volume occupied by micelles, with the formation of more closely packed gel causing rapid gelation at lower temperatures^9, 38^.

Based on the previous results, formulations (F8-F10) were further considered for combination with the mucoadhesive agents. When CS was used as a mucoadhesive agent in our *in situ* gelling systems (F14-F22), precipitation of DMH was observed, which may be attributed to the acidity imparted to the formulations by the addition of CS solution dissolved in 0.1 N acetic acid (pH 2.9), which inhibited the solubilization of dimenhydrinate hydrochloride salt. Hence, CS was excluded as mucoadhesive agent for our systems. As a mucoadhesive agent, HA at a concentration of 1.5% in the formula (F23) resulted in a very viscous and unpourable formulation, most probably because hydrogen bonds between carboxyl groups of HA and poly(ethylene oxide) (PEO) blocks of the poloxamer molecules may have lowered the hydrophilicity and solubility of the poloxamer molecules. Upon decreasing its concentration to 0.5% and 1%, pourable viscous liquids were obtained. Upon testing the gelation temperatures for *in situ* gelling systems containing HA, only formulae (F26-F29) showed sol-gel transition temperatures in the physiological range as shown in Table (1), and hence, formulae (F24) and (F25) showing gelation at 39 ± 2 °C and 38 ± 1.8 °C respectively were excluded from further characterization experiments. Noteworthy that with the exception of *in situ* gels formulated using poloxamers P407:P188%w/w of ratio 20:11%w/w, the addition of HA at 0.5% as in formulae (F24 and F26) significantly increased the gelation temperature (P < 0.05), compared to their counterparts not containing HA (F8 and F9) respectively. As also noted with the non-mucoadhesive formulae (F7-F10), the increase in the ratio of poloxamer P407 decreased the gelation temperature in the mucoadhesive formulae from 39 ± 2 °C (F24) with P407/P188 ratio (18/13) to 35 ± 2.5 °C (F26) with P407/P188 ratio (19/12) then was decreased to 29.4 ± 0.5 °C in (F28) with P407/P188 ratio (20/11).

### Preparation of DMH loaded PEVs

PEVs were prepared using the reversed phase evaporation technique (REV) using soybean phosphatidylcholine as bilayer forming lipid. The choice of the method was based on the fact that REV was more suited for encapsulation of hydrophilic drugs, such as DMH with a log P −0.39^46, 47^.

The selected penetration enhancers to be incorporated in the vesicles were labrasol, transcutol and PEG 400. Labrasol (capryl-caproyl macrogol 8-glyceride) was reported to possess a tight junction opening action leading to increased membrane permeability for water-soluble drugs^48^. On the other hand, transcutol (diethylene glycol monoethylether) is a strong solubilizer with low toxicity, which was found to enhance the intranasal bioavailability of drugs^49, 50^. PEG 400 was incorporated in the hydrating buffer for the solubilization of DMH, and it was reported to enhance the intranasal penetration of drugs by increasing the vesicular bilayer fluidity, thus facilitating the penetration of the fluidized vesicles^4^.

### Determination of DMH EE% in the PEVs

The EE% results of the prepared PEVs (V) and (P) were 82.95 ± 3.21% and 84.36 ± 0.55% respectively. These high EE% values could be attributed to the hydrophilic nature of DMH (log p = −0.39), which allowed its incorporation in the large hydrophilic core of the PEVs created by the reverse phase evaporation technique, which results in a high aqueous space-to-lipid ratio and increases the capability of the vesicles to entrap a large percentage of hydrophilic drugs^46, 47, 51^. The penetration enhancers may have also contributed to the high EE% of the drug in the aqueous core, owing to their hydrophilic natures^32^.

### Determination of the particle size, PDI and zeta potential of PEVs

As shown in Table (3), the particle size of the prepared PEVs (V) and (P) were 121.3 ± 9.27 nm and 196.2 ± 29.35 nm. The significantly higher particle size of formula (P) compared to formula (V) could be attributed to their higher content of phospholipids^52^. Formula (V) showed a significantly lower PDI value (0.251 ± 0.07) than formula (P) (0.75 ± 0.05), which might be attributed to the enhanced interaction between the aforementioned enhancers and the phospholipid bilayers upon increasing their amounts, resulting in alteration in the homogeneity of the obtained vesicular population. Both formulations showed a near neutral surface charge (0.83 ± 0.57 and 0.64 ± 0.82 mV respectively) owing to the non-charged nature of the utilized penetration enhancers^53^.

**Table 3:** Effect of storage on the stability of the PEVs.

Formula Code	P.S. of thefreshly prepared PEVs (nm)	P.S.of PEVs after 3 months storage (nm)	Zeta potential of the freshly prepared PEVs (mV)	Zeta potential of PEVs after 3 months storage (mV)	PDI of the freshly prepared PEVs	PDI of PEVs after 3 months storage
**V**	121.3 ± 9.27	136.3 ± 3.5	0.83 ± 0.57	0.74 ± 0.01	0.251 ± 0.07	0.221 ± 0.03
**P**	196.2 ± 29.35	221.5 ± 18.3	0.64 ± 0.82	0.69 ± 0.72	0.75 ± 0.05	0.66 ± 0.2

### Stability study

Upon storage of the formulations for three months in the refrigerator, they showed an insignificant difference in their properties (p > 0.05) as shown in Table (3), indicating their stability.

### Determination of the morphology of formula (V) using TEM prior to incorporation in the *in situ* gelling system

**Figure 2:**
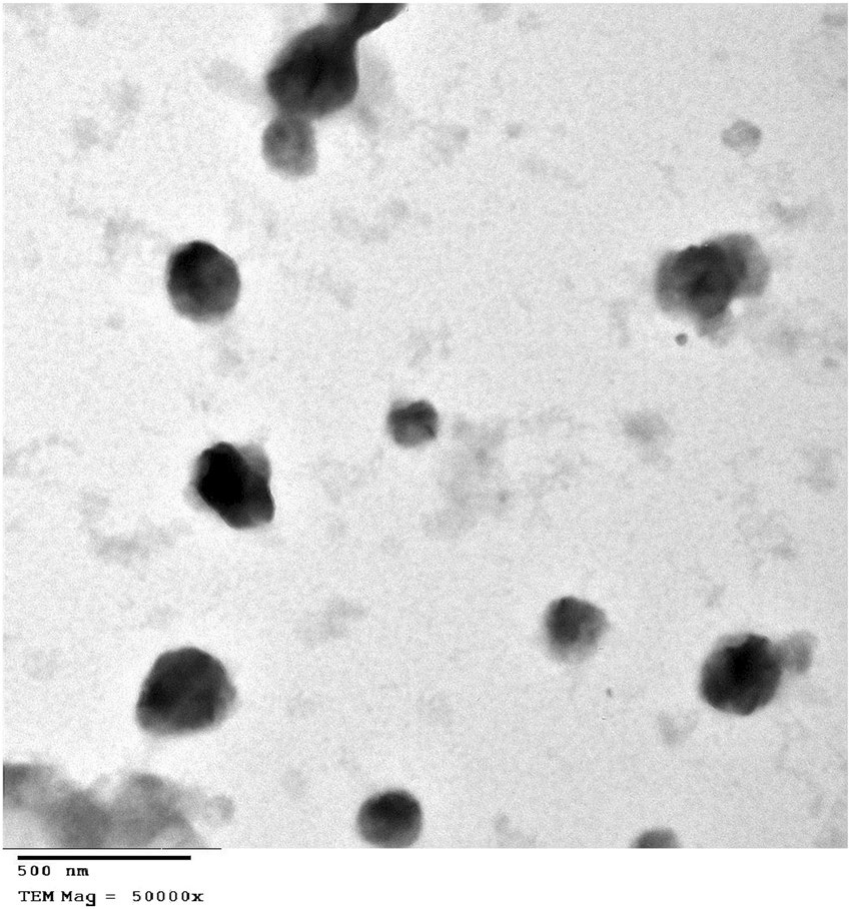
Transmission electron micrograph of the PEVs formula V taken at a magnification of 50000X.

As formula (V6) displayed the best gelation properties (to be shown in the following section), its corresponding PEVs formulation (V) was chosen for morphological examination. The electron micrograph is displayed in Fig. (2), showing spherical vesicular structures.

### Characterization of DMH nanocomposite *in situ* forming gelling systems

#### Measurement of the sol–gel transition temperatures (Tsol–gel) of the nanocomposite *in situ* forming gels of DMH

After conduction of preliminary gelation studies, the proposed combination of poloxomer 407 (P407) and poloxomer 188 (P188) was 19% and 12% respectively, with HA concentration of 1% (F27), since this combination exhibited gelation at 34 ± 0.5 °C which lies in the physiological range. The prepared formulations (V) and (P) were further incorporated in the proposed thermosensitive *in situ* gelling system. However, by incorporating formula (P) prepared using 900 mg phospholipids in the *in situ* gel of P407 and P188 ratios 19% and 12% respectively yielding formula (P1), the gelation temperature was increased dramatically above the physiological required temperature as shown in Table (2). This came in contrast to what was reported by Nie *et al*.^54^ who indicated that the incorporation of liposomes in the *in situ* gel system caused a decrease in the gelation temperature. Therefore, a change of these concentrations was deemed necessary to achieve a suitable sol-gel transition temperature. In an attempt to decrease the gelation temperature to reach the physiological range, single poloxamer type either P188 or P407 was used at concentration of 31% as Yong *et al*.^24^ indicated that the combination of poloxamers P188 with P407 led to an increase in the gelation temperature. However, this either yielded a very viscous gel (formula P2), or no gelation at the physiological temperature (formula P3). On the other hand, upon incorporation of poloxamers P407 and P188 at the ratio of 19% and 12% respectively with formula (V) prepared using 600 mg phospholipids yielding formula (V1), a gelation temperature below the physiological temperature was observed (29.33 °C ± 0.58). This unexpected increase in gelation temperature obtained with the high phospholipid amount (900 mg) in formulae (P) compared to the relatively lower phospholipid amount (600 mg) in formula (V) implies some sort of interaction between the phospholipids and poloxamers^55^, probably occurring at high phospholipid:poloxamer ratios, which in turn affects the gelation temperature. Based on the previous findings, further optimization of the poloxamer P407:P188 ratio on formulae prepared using 600 mg phospholipids was done, in order to achieve a desirable gelation temperature. The progressive decrease in poloxamer P407 ratio compared to P188 (formulae V2-V4) led to an increase in the gelation temperature above the physiological temperature, which was reported to be caused by the lower number and volume occupied by micelles at higher temperatures, with more closely packed gel structure as the concentration increases^56, 57^. Therefore as evident from Table (2), the optimum gelation temperature was expected to occur somewhere between the ratio 19:12 and 18:13, therefore formulae (V5) and (V6) with P407:P188 ratios of 18.5:11.5 and 18.75:11.25 were prepared, yielding gelation temperatures of 32.83 °C ± 1.04 and 35.67 °C ± 0.29 respectively. Formula (V6) was chosen for the next characterization steps as it displayed the most favorable gelation temperature among other formulae.

### Measurement of viscosity of selected formulations

The viscosity of the selected nanocomposite *in situ* gel formulation (V6) and its corresponding PEVs formula (V) was measured both at 25 °C representing the room temperature and 37 °C representing the body temperature. As evident in Table (4), the viscosity of formula (V) was non-significantly decreased upon increasing the temperature to 37 °C (P > 0.05). In case of the nanocomposite formula (V6), the measured viscosity at 25 °C was significantly higher than that of formula (V) owing to the presence of poloxomer and HA polymers (p < 0.05). The increase in temperature to 37 °C resulted in a significant increase in viscosity of (V6), which further proved the formation of gel at the physiological temperature caused by the thermosensitivity of the poloxomer polymer (P < 0.05).

**Table 4:** Viscosity values of the selected PEVs formula compared to the composite form.

Formula code	Viscosity (Pa.S)	
	at 25 °C	at 37 °C
V	0.0124 ± 0.0003	0.0085 ± 0.0003
V6	2.13 ± 0.04	3.2267 ± 0.0153

### *In vitro* release of DMH from selected formulations

**Figure 3:**
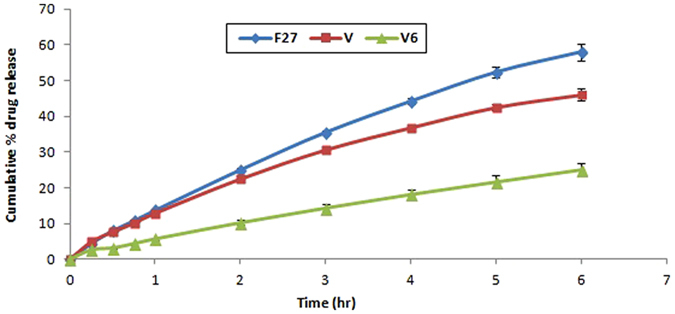
*In vitro* release profiles of the PEVs formula V compared to its nanocomposite counterpart V6 and its gel counterpart (F27).

The cumulative percentage of DMH released from the selected nanocomposite *in situ* gel formulation (V6) and its corresponding PEVs formula (V) and gel counterpart (F27) was determined by applying dialysis in phosphate buffer (PB pH 6.8) at 37 °C over a 6 hours period. Release profiles are demonstrated in Fig. (3). The cumulative percentage of DMH released from the PEVs formula (V) was 12.94% after the first hour and 46.11% at the end of the 6 hours owing to the encapsulated nature of the drug within the PEVs, allowing its sustained diffusion across the vesicular structures. Similarly, the percent of drug released from the gel formula F27 was 13.74% after the first hour and 58.04% after 6 hours, which is slightly larger than the values encountered with formula (V). On the other hand, the nanocomposite formula (V6) showed percentage of drug release of 5.71% after the first hour and a percentage of 24.99% after 6 hours. The cumulative percent released from the latter was significantly lower than the former formulations (P < 0.05) owing to the double encapsulation of DMH first in the vesicles and second within a viscous *in situ* gelling matrix caused by the presence of the thermosensitive polymer poloxomer (P407 and P188)^17, 39^, and the presence of the mucoadhesive polymer HA.

The drug was released from the PEVs formula (V) by a diffusion controlled mechanism, which came in accordance with Nasr *et al*.^28^. On the other hand, the drug was released from (V6) following zero order kinetics. This came in accordance with Nie *et al*.^54^; who reported that the prepared liposomal drug *in situ* gel system displayed zero-order mediated release of the drug. This further highlights the controlled release conferring properties of our prepared nanocomposite system.

### Measurement of the mucoadhesive strength of the selected formulations

The mucoadhesivness of both formula (V) and the nanocomposite formula (V6) was measured using the texture analyzer. Results showed higher mucoadhesive force of the nanocomposite *in situ* gelling system (V6) compared to the PEVs formula (V), in which formula (V6) showed a peak attachment of 0.62 ± 0.13 N with a corresponding work of adhesion 3.23 ± 1.15 mJ, while (V) showed a significantly lower peak attachment of 0.07 ± 0.04 N and a corresponding work of 0.017 ± 0.06 mJ (P < 0.05). This could be attributed to the poloxamer and HA content of the former, which allow increased bioadhesion to mucin^58^.

### Stability of the selected *in situ* gelling formula

After storing the selected formulae **(**V6) in the refrigerator for three months, its sol-gel transition temperature was re-measured. The storage caused a slight decrease in the gelation temperature of formula (V6) to 34 ± 0.5 °C compared to the fresh one which showed gelation temperature of 35.67 ± 0.289 °C (P < 0.05). However, this decrease in the gelation temperatures was still in the physiological range. Furthermore, the formula preserved its clarity over the storage period.

### Pharmacodynamic study using a chemotherapeutically induced emesis model

The cisplatin injection (group II) caused significant reduction in food and water intake compared to the standard normal group (group I) (p < 0.05), while causing a significant increase in kaolin consumption (p > 0.05) as previously predicted by Liu *et al*.^43^. This confirmed the validity of model induction using cisplatin. Upon consumption of a toxic substance; cisplatin in our case, rats tend to dilute the effect of the toxin by consumption of a clay like material such as kaolin (Pica effect) to reduce its adverse effects, similar to the emetic response in humans^59^. Pica effect was reported as a defense mechanism exhibited by rodents (including rats) that lack the emetic reflex to get rid of ingested toxic substances. This is attributed to the fact that clays like kaolin may alter GI transit and absorption or acts by binding or diluting a toxin in the GI tract to reduce its adverse effects^59^. This resembles the emetic response in human beings, which acts as a primarily protective reflex in response to the ingestion of toxic compounds. Moreover, it is also suggested that the gastric dysmotility (decrease in gastric emptying and induction of gastric distension) underlies the alterations in feeding behavior (decrease in food ingestion, increase in kaolin intake).

Table (5) shows the average daily food intake of rats before and after induction for all groups. It was clear that after induction, groups III, IV, V and VI showed a significant increase in food intake compared with the cisplatin control (group II) (p < 0.05). Dramenex® increased the food intake to 10.69 ± 0.36 gm, while (V), (F27) and (V6) increased it to 9.24 ± 0.27 gm, 10.93 ± 0.37 gm and 12.69 ± 0.48 gm respectively compared to the cisplatin control (6.57 ± 0.32 gm) (p < 0.05). Upon comparing groups IV-VI which were administered our selected formulae, the increase in the food intake was in the following order (V6) > (F27) > (V); which demonstrates that the intranasal administration of the *in situ* gelling systems (F27 and V6) was more effective than the administration of mere vesicles (V), with V6 allowing significantly higher food intake compared to F27 (p < 0.05). By comparing the efficiency of the selected formulae with the oral (Dramenex®), it can be observed that formulae V6 displayed significantly higher food intake than that encountered with the oral (Dramenex®), despite being given at a lower dose, suggesting that it more efficient than the former.

**Table 5:** Behavior of different rat groups in terms of food intake, water consumption, kaolin intake and stomach weight content.

Groups			Food intake (gm) Mean ± S.E n = 8		Water consumption (ml) Mean ± S.E n = 8		Kaolin intake (gm) Mean ± S.E n = 8		Stomach weight content (gm) Mean ± S.E n = 8
			Before induction	After induction	Before induction	After induction	Before induction	After induction	After decapitation
Group I	Normal control	Distilled water	18.79 ± 0.48	19.25 ± 0.80^*^	26.75 ± 1.95	26.38 ± 1.8^*^	0.41 ± 0.03	0.34 ± 0.02^*^	2.856 ± 0.21^*^
Group II	Cisplatin control	Cisplatin	18.91 ± 0.51	6.57 ± 0.32^#^	28.63 ± 0.93	14.88 ± 0.69^#^	0.40 ± 0.02	4.05 ± 0.39^#^	10.05 ± 0.48^#^
Group III	Dramenex®	Oral tablets	18.78 ± 0.63	10.69 ± 0.36^*#^	27.5 ± 0.96	22.13 ± 0.55*	0.39 ± 0.38	0.47 ± 0.01*	8.23 ± 0.22^*#^
Group IV	V	Selected PEVs	19.09 ± 0.62	9.24 ± 0.27^*#^	25.38 ± 1.05	19.88 ± 1.17^*#^	0.38 ± 0.04	0.46 ± 0.03^*^	9.573 ± 0.4413^#^
Group V	F27	Selected *In situ* gel	19.42 ± 0.53	10.93 ± 0.37^*#^	25.00 ± 1.13	23.13 ± 0.7^*^	0.39 ± 0.04	0.44 ± 0.03^*^	7.099 ± 0.38^*#^
Group VI	V6	Nanocomposite*in situ* gel (PEVs + *in situ* gel)	19.28 ± 0.54	12.69 ± 0.48^*#^	25.63 ± 1.10	22.25 ± 0.68^*^	0.40 ± 0.02	0.41 ± 0.04^*^	6.628 ± 0.1448^*#^

^#^Significantly different from normal control. ^*^Significantly different from induced group.

The water consumption for rats of different groups is also demonstrated in Table (5). As evident from the results, groups III-VI showed significantly higher water consumption (22.13 ± 0.55 ml, 19.88 ± 1.17 ml, 23.13 ± 0.79 ml, 22.25 ± 0.68 ml) for groups III, IV,V,VI respectively, compared to group II (14.88 ± 0.69 ml) (p < 0.05). Upon comparing groups IV-VI, their effect on water uptake can be arranged in the following decreasing order (F27) > (V6) > (V). However, the difference between them was found to be statistically insignificant (p > 0.05). Similarly, upon comparing water consumption values for the three formulae to the marketed Dramenex® the difference in values was found to be statistically insignificant (P > 0.05), despite being administered at a lower dose, suggesting their effectiveness.

Kaolin; which is a substance of no nutritional value is not normally eaten by rats. Therefore, it was logic to find the results of the daily kaolin intake by the rats of all groups very low ranging from 0.38 ± 0.04 gm to 0.41 ± 0.03 gm under normal conditions (without cisplatin injection), as shown in Table (5). Group II rats that were injected cisplatin i.p. (after induction) showed large increase in the daily kaolin consumption from 0.40 ± 0.02 gm to 4.05 ± 0.39 gm. This increase was significantly higher than the daily kaolin intake of the normal control group (p < 0.05). Treating the rats with the selected formulae and the marketed product (Dramenex®) prior to cisplatin injection significantly decreased the daily consumption of kaolin in comparison with cisplatin control group (p < 0.05). Rats of groups III-VI showed significantly lower kaolin consumption (0.47 ± 0.01 gm, to 0.46 ± 0.03 gm, 0.44 ± 0.03 gm and 0.41 ± 0.04 gm) respectively, compared to rats of (group II) showing 4.05 ± 0.39 gm (p < 0.05). All formulae showed insignificant kaolin consumption compared to Dramenex® (p > 0.05), but at a lesser dose.

Rats expressing the pica response also showed an increase in the wet weight of the stomach content^60^. The gastric retention of solid material has been reported as novel indicator for predicting the potential of compounds that induce pica in non-vomiting rodents^61^. Therefore, at the end of the experiment (24 hours post-cisplatin injection), the whole stomach contents for the rats of the six groups were collected and weighed separately. As shown in Table (5), the average stomach content weight of group I (standard normal) was 2.856 ± 0.21 gm. The cisplatin injection showed a significant increase in the stomach content weight to 10.05 ± 0.48 gm in rats of group II (cisplatin control) (p < 0.05). Groups III, V and VI showed a significant decrease in stomach weight content compared to group II (p < 0.05), while group IV didn’t show a statistical difference from group II (p > 0.05).

The nanocomposite formula displayed better pharmacodynamic performance than PEVs owing to their increased mucoadhesiveness, which allows more DMH release *via* the nasal mucosa. The intranasal delivery of DMH offered by the nanocomposite formula (V6) most probably has delivered the drug to the brain *via* the nanometer sized vesicles through both the general circulation after being absorbed *via* the large nose vasculature and by directly targeting the brain *via* the olfactory epithelium, which offers a direct pathway between the nasal cavity and the brain. This would solve the problem of the first pass effect experienced in the oral route, and achieve a faster onset of action. through direct targeting of the brain *via* the olfactory epithelium^62–64^.

### Histological study of the selected formulations

**Figure 4:**
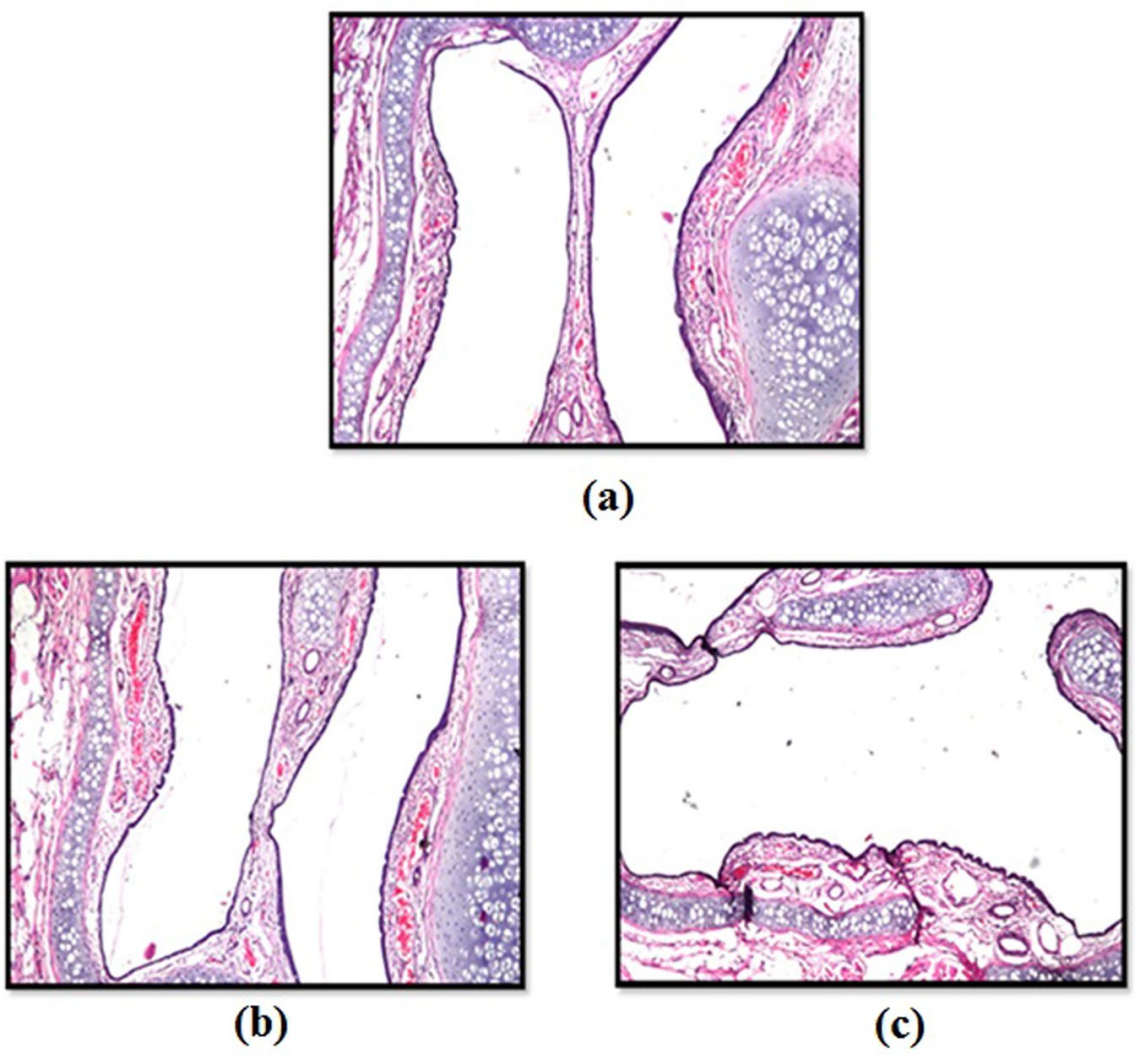
Photomicrographs of the anterior cross sections of control rat nasal cavity (**a**) compared to those administered the *in situ* gelling formula F27 (**b**) and the nanocomposite formula V6 (**c**).

Figure (4) represents photomicrographs taken from the anterior cross sections of the standard control rat nasal cavity (Fig. 4a) compared to rats administered the *in situ* gelling formula (F27) (Fig. 4b) and the nanocomposite formula (V6) (Fig. 4c). The nasal mucosa and underlying cartilage of the rats after being treated with both formulae for 24 hours were found to exhibit normal histological structure with no severe signs such as sloughing of epithelial cells, necrosis or hemorrhage. However, mild congestion in lamina propria of the mucosal lining epithelium was noticed in the tested nasal mucosa of rat treated with the DMH *in situ* gel (F27) as shown in Fig. (4b), which further depicts the merits of encapsulation of active ingredients within vesicles before direct inclusion in gels, since they shield the nasal mucosa from the direct irritant effect that may be caused by the close attachment of gel to the nasal mucosa.

## Conclusion

The combination of different materials within the same system, with the filler being in the nanometer range confers desirable properties to the nanosystem. The current work proves the applicability of nanovesicular incorporation within *in situ* gelling system composed of two polymers in the successful intranasal delivery of the antiemetic dimenhydrinate, provided by the nanosize and penetration enhancing ability of the vesicles and the mucoadhesiveness and viscosity of the *in situ* gelling system. This provides opportunities for more interesting combinations.
